# Pneumococcal Meningitis and its Sequelae – A Devastating CNS Disease

**DOI:** 10.34763/jmotherandchild.2020241.2010.000009

**Published:** 2020-07-29

**Authors:** Monika Bekiesińska-Figatowska, Agnieszka Duczkowska, Marek Duczkowski, Hanna Brągoszewska, Jarosław Mądzik, Beata Iwanowska, Anna Romaniuk-Doroszewska, Dorota Antczak-Marach

**Affiliations:** 1 Department of Diagnostic Imaging, Institute of Mother and Child, Warsaw, Poland; 2 Clinic of Neurology of Children and Adolescents, Institute of Mother and Child, Warsaw, Poland

**Keywords:** Streptococcus pneumoniae, meningitis, neurological sequelae, brain, magnetic resonance imaging

## Abstract

**Introduction:**

In countries where Haemophilus influenzae type B vaccine is used, Streptococcus pneumoniae is the most common cause of bacterial meningitis in young children and notable cause of morbidity/mortality. The authors present material of magnetic resonance imaging (MRI) of patients with pneumococcal meningitis from archive of Department of Diagnostic Imaging of Institute of Mother and Child in Warsaw.

**Materials and methods:**

We performed 27 brain MRI scans and 1 follow-up computed tomography (CT) in 10 children (2 girls and 8 boys) aged from neonate to 5 years at disease onset with proven pneumococcal infection.

**Results:**

Follow-up period range was 0–12 years. Two children underwent only one MRI, one of them died before follow-up and the other was lost from further observation. There was one case of relatively benign disease course with mild changes on MRI. In another seemingly benign case, acute transient hydrocephalus was observed. Six children developed hydrocephalus, and two required ventriculoperitoneal shunting complicated by neuroinfection, shunt malfunction and revisions. Two patients developed epilepsy. In six children, spastic paresis of various severity was diagnosed, up to quadriplegia in one who is under the longest observation (>12 years) and survived in vegetative state. Three other children suffer from delayed psychomotor development to severe intellectual disability.

**Conclusions:**

MRI shows perfectly the degree of central nervous system (CNS) damage during and after pneumococcal invasion. Despite appropriate treatment, disease course may be unpredictably serious. Attempts to eliminate the obligation to vaccinate are extremely irresponsible taking into account potential danger of death, vegetative state or another form of severe damage to CNS. Social and financial costs of care of survivors are very high with shunts placements and changes, (neuro)infections, rehabilitation, families breakdown, etc.

## Introduction

In countries where the *Haemophilus influenzae* type B vaccine is widely used, *Streptococcus pneumoniae* is the most common cause of bacterial meningitis in young children (1). Invasive pneumococcal disease (IPD) is diagnosed when *S. pneumoniae* bacteria are isolated from blood or cerebrospinal fluid. Despite the availability of antibiotics, IPD is a notable cause of morbidity and mortality worldwide. At times of irresponsible attempts to liquidate the mandatory preventive vaccinations in the name of ill-conceived freedom, it is particularly important to remember about the potential devastating results of the diseases that one can be prevented from due to vaccination.

Thoughtless anti-vaccination movements pose a threat to societies because the success in the fight against infectious disease is determined by the percentage of those vaccinated in the society for ‘group immunity’ to occur. This percentage depends on a coefficient called the basic reproduction number (*R*_0_) of an infection and it reflects the number of cases; one case generates on average over the course of its infectious period, in an otherwise uninfected population. If *R*_0_ > 1, the infection will be able to spread in a population. The values for *R*_0_ estimated in several countries over the world for pneumococcal infection range between 1.2 (Hong Kong) and 4.7 (Norway) and resemble *R*_0_ estimates for other respiratory pathogens such as influenza (2), and the median *R* value for seasonal influenza is 1.28 (3).

After the introduction of heptavalent pneumococcal conjugate vaccine (PCV7), the rates of pneumococcal meningitis in the United States declined significantly by 30% in general population and the greatest decline was seen among children below 2 years of age (decrease by 64%) and people older than 64 years (decrease by 54%) (4). The Spanish authors described the impact of the 13-valent PCV (PCV13) on IPD in children younger than 15 years: after 6 years of the inclusion of PCV13 in the vaccination calendar, the aggressive, resistant and most prevalent serotype in their population (19A) almost totally disappeared which led to marked decline in the incidence of meningitis (5).

Pneumococcal meningitis belongs to potentially devastating diseases with high mortality rate (16–37% in adults) and severe neurological damage among survivors (30–52%) (6). Some authors reported intracranial complications rate as high as 74.7% in adults (7). In children the prognosis is slightly better; however, additionally hearing sequelae are present in up to 30% of survivors (6). Nevertheless, the neurological sequelae of IPD were reported in 20–40% of children at the time of hospital discharge before the advent of PCV (1). The risk of sequelae is six times higher in IPD than in other bacterial meningitis (6).

The authors presented the material of magnetic resonance imaging (MRI) of patients with pneumococcal meningitis from the archive of the Department of Diagnostic Imaging of the Institute of Mother and Child in Warsaw. Bioethical Committee approval was waived due to the retrospective character of the study. This presentation of the clinical and radiological appearances of IPD constitutes a part of the current activity of the Institute in the field of public health and aims to get involved in the Institute's activities in the field of ‘Immunization – how to encourage the unconvinced’.

## Materials and methods

We performed 27 brain MRI scans and 1 follow-up computed tomography (CT) scan in 10 children (2 girls and 8 boys) with proven pneumococcal infection, aged from neonate to 5 years at the moment of disease onset.

## Results

The neuroimaging findings in our patients and follow-up data are presented in Table 1.

**Table 1:** Neuroimaging findings in patients with pneumococcal involvement of the brain in our study group

**Patient No.**	**Sex**	**Age at first scan**	**No. of scans**	**Age at last scan**	**Follow-up period on MRI**	**Meningeal enhancement**	**Meningeal empyema(s)**	**Vasculitis with brain infarcts**	**Cavities**	**Hydrocephalus**	**Surgical, neurological and developmental sequelae**
1	M	1st month	3	11 years	11 years	**+**	**−**	**−**	**−**	**+**	Epilepsy; CP (marked quadriplegic paresis); severe intellectual disability; PEG; lying obese patient; severe scoliosis; history of aspiration pneumonias
2	M	4/12	1	**−**	0	**+**	**−**	**−**	**+**	**−**	Died
3	F	6/12	1	**−**	0	**+**	**+**	**+**	**+**	**+**	8 months post-IPD: delayed psychomotor development, left peripheral facial paresis, reduced muscle tone, discrete left hemiparesis, does not focus the eyes
4	M	7/12	2	31/12	2 years	**+**	**−**	**+**	**+**	**−**	4 years post-IPD: epilepsy with focal seizures, CP (mild right hemiparesis)
5	M	8/12	5	6 years	5 years	**+**	**+**	**+**	**−**	**+**	6 years post-IPD: reduced muscle tone, discrete paresis of left hand, severe intellectual disability; history of ventriculoperitoneal shunt and revisions
6	M	13/12	2	14/12	4 weeks	**+**	**+**	**−**	**−**	**−**	lost from further follow-up
7	M	15/12	3	19/12	4/12	**+**	**−**	**+**	**+**	**+**	lost from further follow-up
8	F	16/12	2	16/12	11 days	**+**	**−**	**+**	**−**	**+**	2 years post-IPD: moderate left spastic paresis leg>hand; history of trepanobiopsy for chronic hygromas–haematomas
9	M	4 years	8	16 years	12 years	**+**	**−**	**+**	**+**	**+**	Coma, then vegetative state; severe spastic quadriplegic paresis; lying patient; history of, ventriculoperitoneal shunt and revisions and of aspiration pneumonias
10	M	5 years	1	**−**	0	**+**	**+**	**+**	**−**	**−**	Lost from follow-up

CP, cerebral palsy; IPD, invasive pneumococcal disease; PEG, percutaneous endoscopic gastrostomy.

The follow-up period ranged from 0 to 12 years.

Two children underwent only one MRI examination, one of them (patient no. 2) died despite treatment before follow-up study and the other (patient no. 10) was lost from further observation. Patient nos. 6 and 7 were also lost from further follow-up after 4 weeks and 4 months, respectively.

Most of our patients were administered ceftriaxone and vancomycin, and one received Amikacin and Unasyn in addition to ceftriaxone. Dexamethasone therapy has been implemented in all patients.

There was only one case of relatively benign course of the disease on neuroimaging (patient no. 6) with only relatively mild changes on brain MRI (meningeal enhancement and empyemas) but the boy was lost from further follow-up. In another seemingly benign case with only small meningeal effusion and a single focal lesion in the callosal splenium that has regressed leaving a tiny cavity after 4 months, transient dilatation of the ventricular system with transependymal transudation was found on a follow-up study and was consistent with acute hydrocephalus (patient no. 7).

Six children developed hydrocephalus, and two of them required ventriculoperitoneal shunting that was complicated by neuroinfection, shunt malfunction and necessity of revisions. Two patients developed epilepsy. In six children spastic paresis of various severity was diagnosed, up to quadriplegia in patient no. 9 who is under the longest observation (>12 years) and survived in vegetative state. Three other children suffer from delayed psychomotor development to severe intellectual disability.

The state of hearing impairment in our patients is currently unknown.

## Discussion

In the course of IPD, bacteraemia itself is not an indication for brain imaging but occurrence of neurological symptoms and no improvement in the patient's condition despite appropriate treatment. This study included all patients with IPD known to authors, who had brain MRI during and/or after the disease. Since the data analysis was carried out retrospectively on the basis of medical documentation, it was not always possible to determine the indications for neuroimaging and patients’ detailed neurological status.

Even in such a small material of 10 patients, the devastating consequences of pneumococcal sepsis are visible. All the children were admitted to our Institute before the introduction of vaccinations against *S. pneumoniae* which are currently obligatory to all babies in Poland since 2017 (10-valent PCV (PCV10) in all the babies born after 31 December 2016).

Antibiotic treatment for IPD typically includes broad-spectrum antibiotics until results of antibiotic sensitivity testing are available. The advanced beta-lactam antibiotics (cephalosporins) are commonly used in combination with other antibiotics to treat meningitis. Most of our patients received ceftriaxone (a third-generation cephalosporin) and vancomycin (glycopeptide antibiotic), and one received Amikacin (aminoglycoside antibiotic) and Unasyn (a combination of the penicillin-derived antibiotic ampicillin and sulbactam, an inhibitor of bacterial beta-lactamase) in addition to ceftriaxone. Dexamethasone was administered in all patients.

Adjunctive dexamethasone therapy has been implemented on a routine basis for patients with suspected or proven pneumococcal meningitis in many countries (8). Corticosteroids significantly reduced hearing loss and neurological sequelae but did not reduce overall mortality in patients of all ages with acute bacterial meningitis in high-income countries (9).

According to Bernson-Leung and Lehman (10), even healthy, vaccinated children can develop IPD, and some studies suggested that *S. pneumoniae* infection is more likely to cause stroke than other pathogens. Among bacterial meningitis, the one caused by *S. pneumoniae* is associated with the highest case-fatality rate (11) that is estimated at about 8% among children and 22% among adults. In the United States, before routine use of PCV, children younger than 1 year old had the highest rates of pneumococcal meningitis. In our material, 50% of patients developed the disease in the first year of life. The pathological (post-mortem) evaluation of adult brains after IPD was conducted and described by Engelen-Lee et al. (12). Meningeal inflammation was present in all their patients, parenchymal brain infiltration of inflammatory cells (cerebritis) in 77%, cerebral infarction in 61%, cerebral haemorrhage in 77%. Fifty-five per cent of patients had both cerebral infarction and haemorrhage, and only 16% had neither infarction nor bleeding. Brain abscess was found in 19% of patients. Inflammation of large-to-medium arteries and the consequent reactive changes were seen in 97% of patients and were associated with the clinical diagnosis of infarction. Arterial thrombosis, thromboembolism and/or sinus thrombosis were observed in 68% of cases. The study by Engelen-Lee et al. showed that vascular damage plays the key role in the process of brain damage in pneumococcal meningitis (12). Cerebrovascular complications of pneumococcal meningitis were attributed to inflammatory infiltrations of cerebral arteries and veins found on autopsies and confirmed by angiographic findings of segmental arterial narrowing in patients with ischaemic stroke complicating pneumococcal meningitis, leading to the conclusion that infarctions in the course of IPD are caused by vasculitis. However, in a study of patients with pneumococcal meningitis, among whom half had cerebral infarctions and 67% microhaemorrhages, no large-vessel vasculitis was found and small-vessel vasculitis was not localised in the areas of infarctions. In these patients, it is suggested that cerebral intravascular coagulation, independent of systemic coagulopathy or cerebral vasculitis, may be the cause of cerebrovascular complications of IPD. So the pathogenesis of cerebral infarctions is still unclear and is the subject of ongoing research (8).

In a Canadian study of stroke in paediatric bacterial meningitis, the mortality rate was 25% in children with stroke compared with 4% in those without stroke. Moreover and obviously, survivors after stroke were more likely to have neurological deficits at follow-up (69%) versus those without stroke (26%) (13).

**Figure 1:**
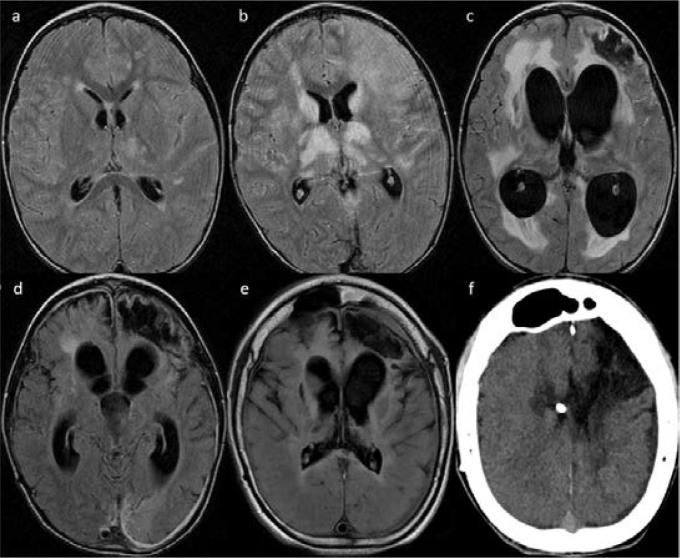
**(A)** magnetic resonance imaging (MRI) at admission. A few ischaemic foci are already observed as FLAIR-hyperintensities in both cerebral hemispheres. **(B)** MRI after 1 week (FLAIR). More extensive, confluent, bilateral infarcts. The biggest one, cortical–subcortical, involves the left frontal lobe. **(C)** MRI 1 month after disease onset (FLAIR). Hydrocephalus with CSF transudation, post-ischaemic lesion in the left frontal lobe with laminar necrosis of the overlying cortex. **(D)** MRI after 7 months (FLAIR). Progression of hydrocephalus, scarring of the right frontal lobe as well, subdural haematoma over the left cerebral hemisphere. **(E)** MRI after >6 years (FLAIR). Smaller degree of ventricular dilatation after ventriculoperitoneal shunt placement. Abnormal, hyperintense white matter with lacunar post-infarct foci. Cortical–subcortical scars in both frontal lobes (L > P). Chronic bilateral subdural haematomas. **(F)** CT after >12 years. Overdrainage with slit-like lateral ventricles. Scar in the left frontal lobe. Thickened calvarial vault associated with ventricular shunting for hydrocephalus at a young age was observed 6 years earlier **(G)**.

In our material, 70% of patients had evidence of infarction on MRI which is consistent with the literature and confirms the severity of IPD (Figure 1).

In an Italian study of 64 children with pneumococcal meningitis before the vaccination era, Pagliano et al. reported the abnormalities on CT scan on admission only in 14 of their patients (22%): brain oedema in 14, arterial infarction in 2 and sinus thrombosis in 2. They scheduled CT at admission and before discharge in all cases. During the hospital stay and the 8-week follow-up period, repeated CT or MRI was scheduled only for patients with either positive findings on admission or with suspicion of cerebral oedema, or in the presence of focal neurological deficit (14).

We hypothesised that some of the abnormalities must have been lost to their attention due to the inadequate neuroimaging method – mostly CT that is considered suboptimal for the detection of sinus thrombosis and early ischaemic stroke in which MRI is the modality of choice with T1, T2, FLAIR, T2* (gradient-echo or susceptibility-weighted), diffusion-weighted and post-contrast sequences, and with magnetic resonance angiography (MRA) and venography, when necessary (15). A good example of suboptimal quality of CT for the detection of neuroradiological findings in our study group is patient no. 5, with two CT scans that did not show any changes, while MRI performed immediately revealed contrast enhancement of the meninges and meningeal empyemas of about 5 mm width. In the material of Pagliano et al., 3% of the children died and 22% showed sequelae, namely paresis, hearing loss, hydrocephalus and cognitive impairment (14), which confirms our opinion about inappropriate imaging method used by these authors. According to the ALARA (As Low As Reasonably Achievable) rule, MRI is a method of choice in neuroimaging in general, in neuropaediatrics in particular. Only on MRI can a cerebral infarction be detected as early as about 30 min after it has occurred due to DWI sequence. Therefore, the choice of a proper imaging method plays a key role in patients’ management. In our Institute, the role of brain CT is limited to the acute cases when MRI unit is not available. However, in another CT-involving study of 28 children with pneumococcal meningitis from pre-vaccination era, 71% of patients required admission to the intensive care unit (ICU), 14% died and on discharge 47% of patients had sequelae: 28% suffered deafness, 14% hemiparesis, 7% severe hydrocephalus and 3.5% mental retardation (16).

In the literature, we found one report concerning cerebellar involvement as a rare complication of pneumococcal meningitis (17). In our material, bilateral cerebellar infarcts were found in two children (patient nos. 8 and 9) and in two other patients (patient nos. 3 and 10) cerebellar atrophy was observed which makes cerebellar abnormalities more frequent complication of IPD than reported.

Even after the introduction of the polyvalent PCV, *S. pneumoniae* still remains the leading cause of bacterial meningitis in children and of the greatest morbidity and mortality: in a study by Sadeq et al. in Kuwait, 48% of children with pneumococcal meningitis were admitted to the ICU, 13% died and 35% were discharged with sequelae (18). As reported by Olarte et al., these numbers in the USA were as follows: 66%, 4% and 39%, respectively (19). In an Australian study 13% of children died, 22% suffered from severe neurological outcomes (paresis, hydrocephalus with shunting, visual loss, marked intellectual impairment) and only 55% recovered without any sequelae (20).

Our study focused on neurological sequelae, and other forms of sequelae (e.g. orthopaedic) were not captured.

Our material demonstrates practically only the complicated cases. With the exception of patient no. 6 who had only relatively mild changes on brain MRI (meningeal enhancement and empyemas) and was lost from further follow-up, others are a tragic illustration of the severity of the disease, its complications and serious consequences for the patient's and his family's further life. Remembering that we have the follow-up data only in 7 out of 10 patients (3 lost from follow-up), our results in terms of lifelong sequelae are dramatic: 1 death, various degree of paresis in 6 patients, numerous neurosurgical procedures in 3, epilepsy in 2, severe intellectual disability in 2 and delayed psychomotor development in 1. In one known case, the child's parents divorced shortly after a very unfavourable prognosis had been established.

In the British material, the pneumococcal meningitis survivors had significantly lower mean full-scale intelligence quotient (IQ), verbal IQ, numeracy, total quality of life, school functioning, psychosocial functioning and greater psychological difficulties than the age-matched controls. Parents of patients reported greater functional disability, lower quality of life and psychological difficulties (21).

## Conclusions

MRI shows perfectly the degree of central nervous system (CNS) damage during and after pneumococcal invasion of the brain. Despite appropriate treatment, the course of the disease may be unpredictably serious. Attempts to eliminate the obligation to vaccinate are extremely irresponsible taking into account the potential danger of death, vegetative state or another form of severe damage to the CNS. The social and financial costs of care of survivors are very high with shunts placements and changes, (neuro)infections, rehabilitation, families breakdown, etc. This publication constitutes part of the mission of the Institute of Mother and Child: ‘Every day we fight for the health, dignity and joy of our patients, remembering what is our greatest value – human life’ with an emphasis on the quality of this life which can be preserved in the case of responsible prevention.
